# Multi-level policy responses to tackle socioeconomic inequalities in the incidence of COVID-19 in a European urban area

**DOI:** 10.1186/s12939-022-01628-1

**Published:** 2022-02-19

**Authors:** Davide Malmusi, M. Isabel Pasarín, Marc Marí-Dell’Olmo, Lucía Artazcoz, Elia Diez, Sara Tolosa, Maica Rodríguez-Sanz, Glòria Pérez, Conchita Peña-Gallardo, Carme Borrell

**Affiliations:** 1 Ajuntament de Barcelona, Barcelona, Spain; 2 CIBER de Epidemiología y Salud Pública (CIBERESP), Madrid, Spain; 3 Agència de Salut Pública de Barcelona, Pl. Lesseps 1, 08023 Barcelona, Spain; 4 Institut d’Investigació Biomèdica Sant Pau (IIB Sant Pau), Barcelona, Spain; 5 Present Address: Universitat Pompeu Fabra, Barcelona, Spain; 6 Consorci Sanitari de Barcelona, Barcelona, Spain

**Keywords:** COVID-19, Inequalities, Public policies, Urban areas

## Abstract

**Background:**

Spain has been hit hard by COVID-19 since March 2020, especially in its metropolitan areas. We share experiences from Barcelona in measuring socioeconomic inequalities in the incidence of COVID-19 in the different waves, and in implementing coordinated and equity-oriented public health policy responses.

**Methods:**

We collected daily data on confirmed COVID-19 cases, geocoded the address of residence to assign each case to one of the 73 neighborhoods and 1068 census tracts, and calculated the cumulative incidence of COVID-19 by neighborhood and five income groups (quintiles of census tracts) by sex across four waves of the pandemic. We adjusted hierarchical Bayesian spatial models to obtain the relative risk (RR) of cumulative incidences in each quintile compared with the richest areas. A variety of public health policies implemented to tackle the pandemic and especially these inequalities in COVID-19 incidence and vaccination are selected and described.

**Results:**

Area-level income inequalities in the incidence of COVID-19 were present at different degree in all four waves. In the second wave (10/1/2020 to 12/6/2020), RR for the poorest income quintile census tracts compared with the richest was 1.43 (95% credible interval-CI-: 1.22–1.67) for men and 1.58 (95% CI: 1.35–1.83) for women. Later, inequalities in vaccination coverage also arose. Equity-oriented policy responses included: “health hotels” or home delivery of basic products for individuals with COVID-19 and without adequate conditions for isolation; new emergency facilities for homeless people, including those with active drug use; mass screening in high incidence areas; contingency plans for nursing homes and schools; adapting community health programs for their early reactivation; digital self-appointment support points and community vaccination days.

**Conclusion:**

COVID-19 hit Barcelona neighborhoods unequally, with variations between waves. The rapid availability of geolocalized data and by socioeconomic level helped public authorities to implement targeted policies and collaborative interventions for the most vulnerable populations. Further studies would be needed to evaluate their impact.

## Background

Spain has been hit hard by the SARS-CoV-2 pandemic, experiencing the largest drop in life expectancy in 2020 in the whole of Europe: 1.6 years [1]. In the first wave, metropolitan areas like Madrid and Barcelona registered very high rates of healthcare system contacts and hospital admissions. The second wave started to emerge early in the summer, driven by a new variant of the virus that later became predominant in Europe.

COVID-19 has spread worldwide and across all levels of society and, as such, has required a population-based and universal approach, involving various levels of governance, such as supranational bodies, country, regional governments and local governments, and community movements. It is at this local level, where specific needs have been identified in all phases of the pandemic. Many of them required rapid responses, equalizing access conditions for socially disadvantaged groups to prevent an increase in social inequalities in health. COVID-19 has shown social inequalities in its incidence, which has been reported to be higher in people with lower socioeconomic position and in ethnic minorities [2].

The city of Barcelona has a long tradition in both the study of socioeconomic inequalities in health and in the development of policy responses to tackle them at the local level. It is also a city with large social contrasts, high levels of poverty in some neighborhoods, and with a highly diverse population, with more than a quarter of residents being born abroad and coming from very different origins [3]. This article aimed to describe the trend in socioeconomic inequalities in the incidence of COVID-19 in Barcelona across its different waves, and to explain different multisectoral, equity-oriented policy responses delivered by public health and municipal services.

## Methods

### Area-based socioeconomic inequalities in the incidence of COVID-19

First, we present the surveillance system set up to monitor area-based socioeconomic inequalities in the incidence of COVID-19. At the start of the pandemic, as part of COVID-19 surveillance, the *Agència de Salut Pública de Barcelona* (ASPB; a consortium between the municipal and regional governments, in charge of public health surveillance, protection and promotion in the city) prepared a website showing daily data by age, sex, neighborhood, basic health area, census tract and census tract income levels (https://webs.aspb.cat/covid19aldiabcn). Here, we present data on Barcelona residents (1,674,903 on 6/30/2019), excluding people living or working in nursing homes because the nursing home address does not necessarily reflect the residents’ socioeconomic position. However, the system enables identification of the impact of the pandemic in nursing homes. We collected daily data on confirmed COVID-19 cases from the Catalan Department of Health. The address of residence (> 98% valid addresses) was geocoded to assign each case to one of the 73 neighborhoods (median population 21,351) and 1068 census tracts (median population 1510). Area socioeconomic status was based on the 2016 personal income index at the census tract level [4]. The number of residents was obtained from the 2019 Municipal census.

We calculated the cumulative incidence of COVID-19 (new cases per 100,000 inhabitants) by neighborhood and five income groups (quintiles of census tracts) by sex in the first four waves (see Figs. 2 and 3 for the range of dates). For each sex and wave, we adjusted hierarchical Bayesian spatial models proposed by Besag, York, and Mollié [5–7] including the average income categorized in quintiles to obtain the relative risk (RR) of cumulative incidences in each quintile compared with the richest areas as the reference group.

### Equity-oriented policy responses to COVID-19

Among a very wide range of local responses to tackle the pandemic and its social and economic consequences, we describe in the Results section nine local interventions selected by the authors considering the following criteria: a) a leading or important collaborative role of public health services; b) including policies that were either: 1) addressed to specific settings or the general population but with an orientation to equity; or (2) directly addressing socioeconomic inequalities or socially deprived populations; and/or (3) specifically designed as a response to socioeconomic inequalities related to COVID-19.

## Results

### Area-based socioeconomic inequalities in the incidence of COVID-19: the data

During the four first waves, 104,447 cases of COVID-19 were diagnosed. The value of the cumulative incidence could not be compared in the four waves because the periods analyzed did not contain the same days, and in the first wave, PCR tests were insufficient and the cases shown were mainly those of hospitalized persons or healthcare workers.

**Fig. 1:**
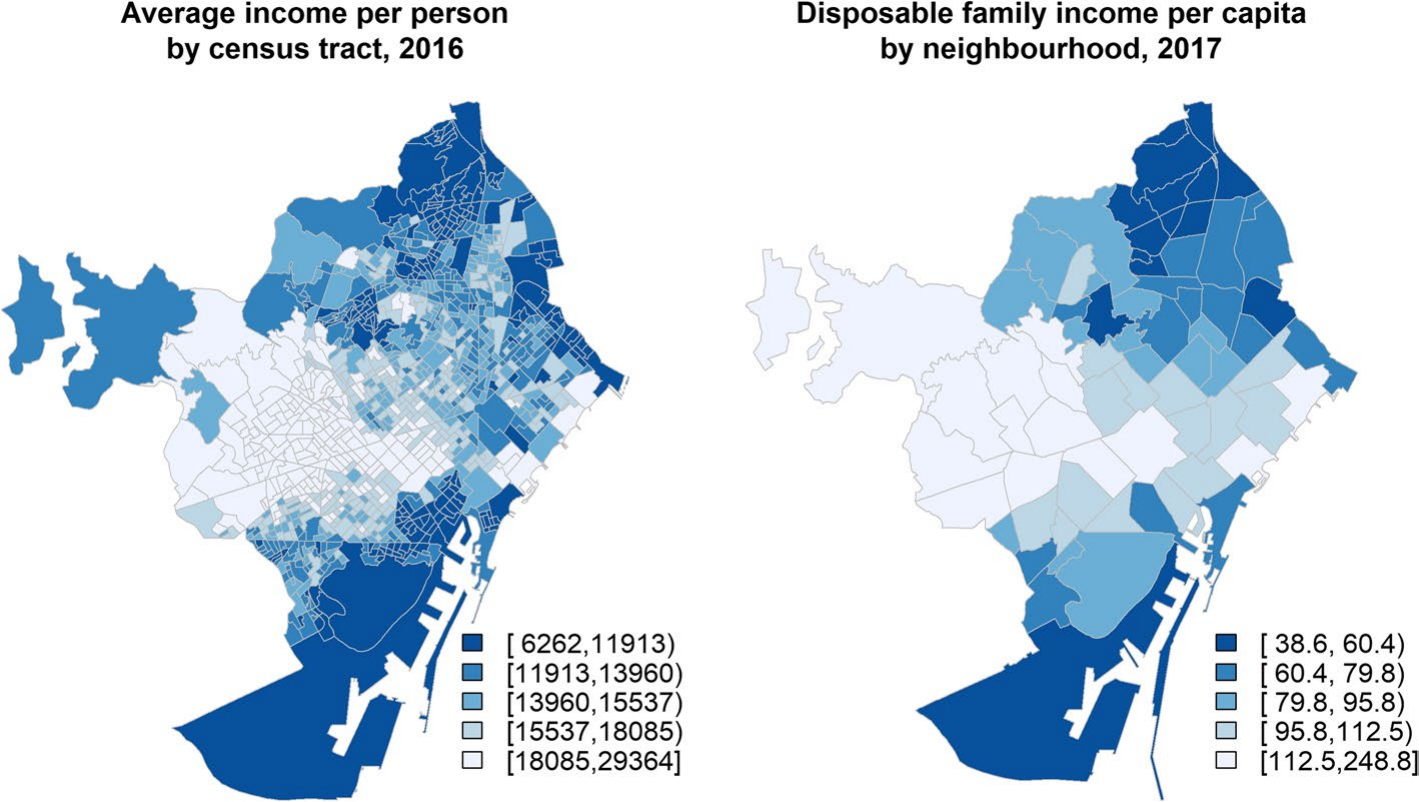
Average income per person by census tract (2016) and disposable family income by neighborhood per capita (2017). Source of average income: https://www.ine.es/prensa/experimental_atlas.pdf. https://inespain.maps.arcgis.com/apps/MinimalGallery/index.html?appid=c8b41b2c471845afbc8f8eb20c54382e#. Source of disposable income per capita: https://ajuntament.barcelona.cat/barcelonaeconomia/ca/renda-familiar/renda-familiar/distribucio-territorial-de-la-renda-familiar-disponible-capita

**Fig. 2:**
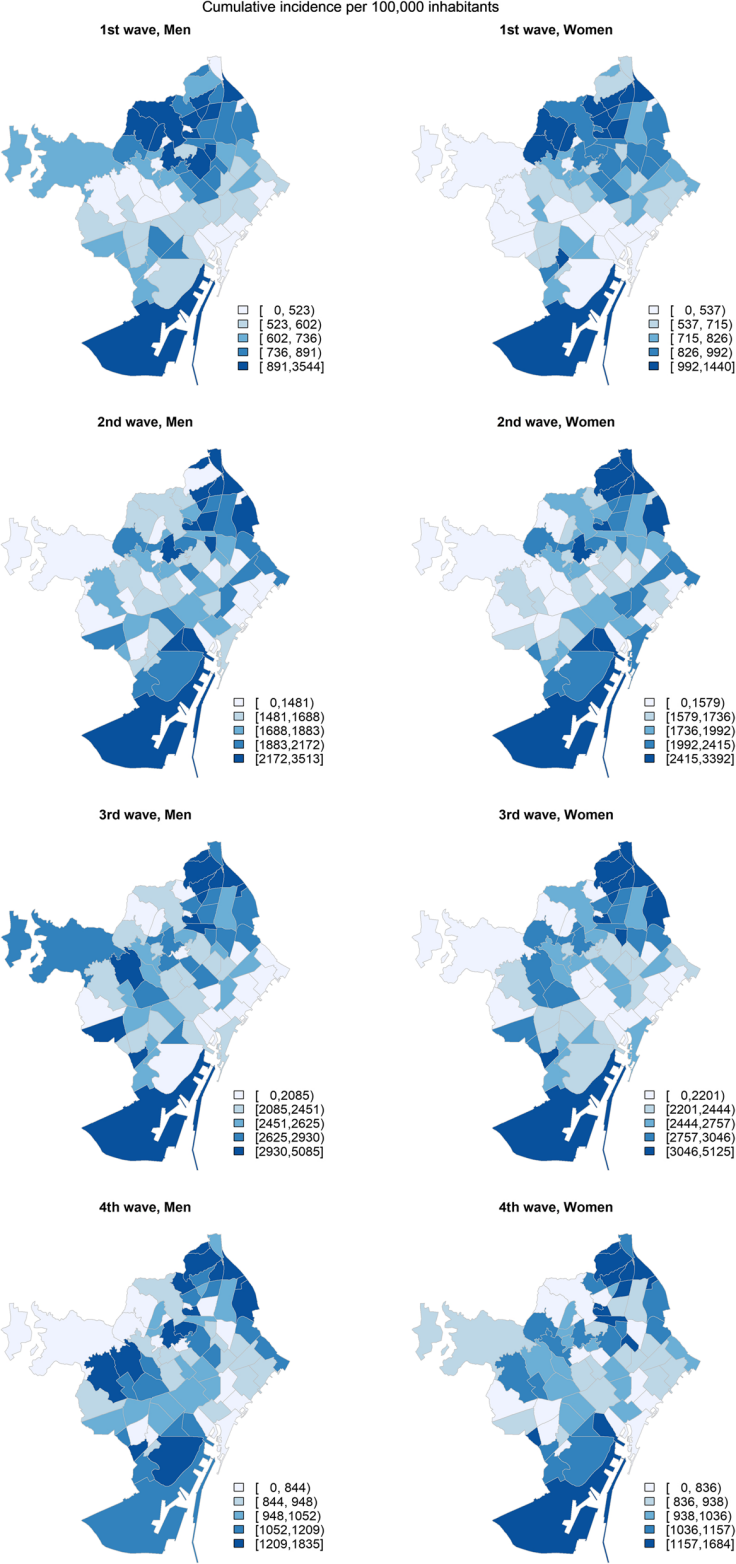
Distribution of cumulative incidence of COVID per 100,000 inhabitants in the neigborhoods for men and women in the four waves. Wave 1: 02/25/2020–07/04/2020 (40 days); wave 2: 10/01/2020–12/06/2020 (67 days); wave 3: 12/07/2020–03/14/2021 (98 days); wave 4: 03/14/2021–05/31/2021 (78 days)

Figure 1 shows the average income per person by census tract, as well as the disposable family income per capita by neighborhood [8]. Figure 2 shows the distribution of the cumulative incidence of COVID-19 by neighborhood for men and women in the four waves. A general pattern of a higher incidence in low-income neighborhoods can be observed.

**Fig. 3:**
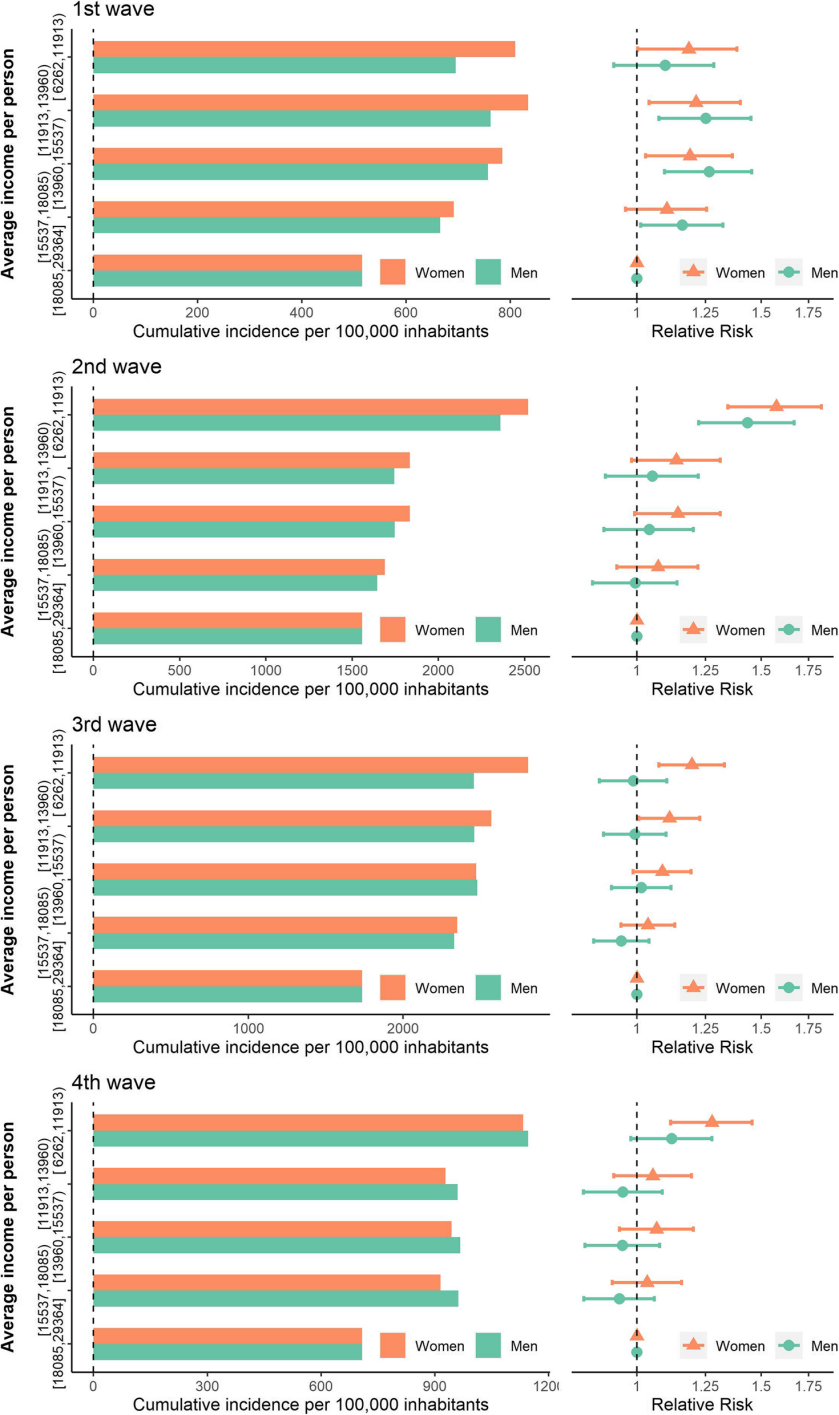
Cumulative incidence and RR by five groups of average income per person at census tract level by sex in the four waves. Wave 1: 02/25/2020–07/04/2020 (40 days); wave 2: 10/01/2020–12/06/2020 (67 days); wave 3: 12/07/2020–03/14/2021 (98 days); wave 4: 03/14/2021–05/31/2021 (78 days)

Figure 3 shows the cumulative incidence by income quintiles (of census tracts), sex and wave, and the RRs of this incidence. Inequalities were more pronounced in the second wave, showing an increasing gradient in the incidence as income decreased. The RR for the poorest income quintile census tracts compared to the richest was 1.43 (95% credible interval-CI-: 1.22–1.67) for men and 1.58 (95% CI: 1.35–1.83) for women. In the third and fourth waves, these inequalities tended to decrease. RRs also showed this pattern and tended to be higher among women than men.

### Equity-oriented policy responses to COVID-19

In the following subsections and in Table 1, the selected nine examples of interventions are described.

1. Policies addressed to a specific setting or the general population but with an orientation to equity

**Table 1:** Selected equity-oriented interventions to tackle the incidence of COVID-19 and inequalities in Barcelona

Intervention	Description	Target population(s)	Activity indicators	Expected or measured impact	Calendar
*Addressed to a specific setting or the general population but with an orientation to equity*					
Interventions in Nursing homes	Activities for the prevention and control of COVID-19 in nursing homes. A joint office was created for management and organization	Residents, workers and administrators in nursing homes	Contingency plans in 229 nursing homes	Control of COVID-19 outbreaks in nursing homes	The office started at the end of March 2020 - present
Interventions in schools	Activities for the surveillance, prevention and control of COVID-19 in schools. Strong support of school directors. Joint office was created for management and organization	Students, workers and administrators in school centers	Only 15% of classes and 7% of students confined to home or quarantined	That the majority of students could attend school in person. Control of COVID-19 outbreaks in schools	9/14/2020–6/22/2021
Communicating risks and containment measures	Risk communication and community engagement strategies to increase the reach of valid information. A website with COVID-19 indicators and with all the public health information and recommendations related to COVID-19.	General population, professionals, decision-makers.	13 press releases, 166 media requests answered and 783 media appearances	958,690 visits vs. 412,225 visits in a comparative period. 76% new visitors.	March 2020 - present
*Directly targeted at addressing socioeconomic inequalities or socially deprived populations*					
Health hotels	Nine hotels converted into accommodation for mild COVID-19 cases, supervised by healthcare personnel	People infected with COVID and with inadequate housing conditions to follow isolation rules	2881 people hosted	Accelerating hospital discharges, cutting transmission chains with cohabitants and community, providing social support to vulnerable populations	March 2020 - present
Quarantine support program	Home delivery of food baskets or cooked meals, personal protective equipment, cleaning and personal hygiene products, home cleaning and disinfection service, waste disposal, dog walking	People infected with COVID and their contacts without resources or social networks	8272 people received food baskets, 948 families received cooked meals	Cutting transmission chains with cohabitants and community, providing social support to vulnerable populations	4/22/2020–7/16/2021
Special facility for homeless people with drug dependency problems	Residential center with harm reduction programs for drug use (supervised consumption spaces and controlled alcohol consumption program)	Homeless people with drug dependency, including alcohol	70 beds; 182 different people have lived in the center to date	Improvement in quality of life of homeless and addicted people. A place for isolation if infected with the disease or becoming a close contact	4/4/2020 - present
Adapting community health interventions to tackle COVID-19	Use the previous structure of the community health network, as well as a communication network, to detect needs or problems during periods of confinement; contact with participants and territorial agents to assess what to do and how; generate responses with community assets to the needs detected; adapt interventions to the “new normal”	Neighborhoods with the greatest socioeconomic deprivation, in which the Barcelona Health in the Neighborhoods program is implemented (25 of the 73 neighborhoods of Barcelona)	13 community programs adapted to pandemic situation	Address determinants of health, such as social isolation, which are worsened by the pandemic	2 stages: first: April 2020–September 2020 Second: October 2020 - to date
*Specifically designed as a response to the appearance of socioeconomic inequalities related to COVID-19*					
Mass screening campaigns in high incidence areas	Opening of free, voluntary PCR testing points in community centers for residents in the whole neighborhood or in selected census tracts, for 2–3 days per point	Four neighborhoods with the highest incidence in the last 15 days	5394 tests, 151 were positive	Cutting transmission chains. Sensitizing residents about the high incidence in the community	8/15/2020–9/20/2020
Vaccination scheduling support points and street points for vaccination without appointment	Universal strategy (online self-scheduling) combined with strategy targeting groups with vulnerabilities (personal support points for online scheduling, and vaccination street points). A joint office was created for management and organization	Neighborhoods with lower vaccination coverage in the first weeks of vaccination	25 support points/1689 users 19 street points/2081 vaccinated	Increase in vaccination coverage. Reduction of the gap between extreme coverages (32 to 15%)	5/26/2021–11/22/2021

#### Interventions in nursing homes

In Barcelona, as in other European cities, the COVID-19 pandemic had a disproportionate effect on nursing homes. As of June 2021, there were 229 nursing homes in Barcelona with 9664 residents and 7400 workers. Since March 2020, there have been 5735 confirmed cases (72.4% women). Differences across Spanish regions in the incidence of COVID-19 and fatalities in nursing homes have been related to underinvestment and traditionally very poor working conditions [9].

In March 2020 a city-level executive board was created with the representation of social services, public health and healthcare services. This group had very frequent in-person and online meetings, which allowed rapid decisions to be taken about difficult issues such as replacing some boards in nursing homes with critical management problems concerning COVID-related mortality, relocating residents, public health interventions for correct sectorization, isolation and quarantines, training of professionals or closing some nursing homes. The ASPB led the development of contingency plans. In June 2020, the board turned into a technical office devoted to COVID prevention and surveillance in nursing homes with about 10 professionals working full- or part-time. Since March 2021, the ASPB has been adapting contingency plans to the vaccination phase. The success of this good practice was largely possible due to the strong commitment of the institutions and the members of the group in a context of extreme complexity. Currently, rethinking the model of nursing homes is on the political agenda.

#### Interventions in schools

In March 13, 2020, schools were closed in Catalonia and did not reopen in the 2019–20 school year. Mandatory schooling from 6 to 16 years is one of the main equity-promoting public policies, with clear repercussions on health inequalities [10], and opening schools on a face-to-face basis was considered a high priority objective for the 2020–21 academic year, even if the pandemic was not under control. In Barcelona, a joint office was created with the main education, health services, public health and municipal managers and technicians, in order to plan and coordinate actions to support schools in implementing measures to control COVID-19 and in maintaining as much in-person activity as possible. During the 2020–21 school year, 15% of classes have been isolated at home or quarantined at some point, but school days have been mostly in-person.

#### Communicating risks and containment measures

As part of the response plans to the pandemic, public health authorities developed risk communication [11] and community engagement strategies to increase the reach of valid information to professionals, decision-makers and the general population, efficiently spread recommended measures, and reduce misinformation [12].

The ASPB created a website with frequently asked questions (FAQs) about COVID-19, available in three languages (Catalan, Spanish and English) and continuously updated. From 18th February 2020 to 27th June 2021, the FAQs have had 283,067 visits, 33% of them in March 2020. Communication actions were continuously coordinated with the Catalan Department of Health, responsible for official public health messages, as well as with the City Council, to disseminate messages as much as possible to the population. The website with COVID-19 indicators (*covid19aldiabcn* explained above) had 136,437 visits from 08/01/2020 to 06/27/2021. Later, a specific website was created with all the public health information related to COVID-19, which also added information and recommendations about environmental and occupational health, food safety, health promotion, etc. The ASPB website received 958,690 visits from 02/18/2020 to 06/27/2021 vs. 412,225 visits in a comparative period before those dates, and a 76% of new visitors. In this period, 13 press releases were issued, 166 media requests were answered and 783 media appearances were registered.

An important strategy was the reinforcement of communicating COVID-19 risk and preventive measures in high-incidence neighborhoods through street-informers, cultural mediators-translators and collaboration with community leaders, using simple messages and formats, informative sessions and popular channels such as WhatsApp.

2. Policies directly addressing socioeconomic inequalities or socialy deprived populations

#### Interventions to support isolation: “health hotels” and “Quarantine support program”

At the beginning of the pandemic, it became evident that there was a need for interventions to help those groups with the greatest difficulty in carrying out isolation and quarantine for COVID-19. Within 2 weeks of the declaration of pandemic, some hotels were converted into “health hotels”, supervised by healthcare personnel and aimed at both accelerating hospital discharges for people recovering from COVID-19 symptoms, and relocating those with mild infections and inadequate housing conditions to follow isolation rules without putting their cohabitants at risk. In the latter case, persons were detected and referred by social workers in primary healthcare centers. From March 2020 to June 2021, 9 facilities were set up and hosted 2881 persons.

However, there was still room to support a wider set of situations with a less intense use of resources: for example, preventing people infected with COVID and their contacts with insufficient economic resources or social networks from going out to buy food or from sharing a kitchen with other families. This was the rationale to allow social workers from the health system to activate the “Cooked Meals at Home” program of municipal social services and, since April, to set up a municipal “Quarantine Support Program”, based on prescription by health system social workerswhich offered home delivery of a food basket (or cooked meals when necessary), personal protective equipment, cleaning and personal hygiene products, a home cleaning and disinfection service, waste disposal, and dog walking. By July 2021, 9902 persons in 2796 apartments had received some kind of service or home delivery from the program.

#### Special facilities and actions for homeless people

In the first 3 weeks of the lockdown, which limited the free use of public space, the City Council prepared a contingency plan to ensure residential and accommodation facilities for homeless people. Social intervention teams matched people sleeping rough to the available facilities and with new centers to receive meals and clothing and to enable showers. In collaboration with social entities, several accommodation facilities were set up: one for 58 chronic homeless people, another with 59 places for women, one with 30 places in single rooms for homeless people with mild Covid-19 symptoms, and two spaces with 225 places in pavilions at the Barcelona Fair. A center with 70 places was also set up for homeless people with addictions to drugs or alcohol and another for 42 young and adolescent male homeless persons [13].

The center for homeless people with drug dependency, both on illegal drugs and alcohol, had, in fact, been included in the city’s “drug action plans” since 2009: the pandemic allowed the opening of such a residential center for the first time in the city, and indeed in the country, with harm reduction services; therefore, the center did not require residents to be abstinent. This allowed initiation of intervention and recovery plans tailored to each hosted individual’s life situation. Of the 70 places provided, a minimum of 25 of them would be reserved for women, and 14 months after opening, 32 places were occupied by women, 33 by men and 1 by a non-binary person.

#### Adapting community health interventions and using community health networks to tackle COVID-19

Another example of addressing inequalities has been the existence of a direct communication link between public health services and local communities to detect and respond to needs, through the community groups created in previous years (for example, the local commissions of the *Barcelona Health in Neighborhoods* program). Above all, we have tried to reactivate community health programs early, and to prevent the social isolation that COVID-19 has entailed from making certain groups (e.g., the elderly) even more vulnerable: to this end, various programs were adapted to the situation implied by COVID-19, most of them with the aim of reducing social isolation in neighborhoods with the lowest socioeconomic level [14].

3. Policies specifically designed as a response to area-level socioeconomic inequalities related to COVID-19

#### Mass screening campaigns in high incidence areas

In summer 2020, COVID-19 cases began to rise again, and their geographical distribution was clearly unequal. Taking advantage from the increased capacity to perform PCR tests, mass screening campaigns were undertaken in order to detect incident cases of COVID-19 in public spaces of several deprived neighborhoods with high incidence. More than 5000 tests were undertaken finding 151 positive cases.

#### Inequalities in vaccination coverage and strategies for their reduction

**Fig. 4:**
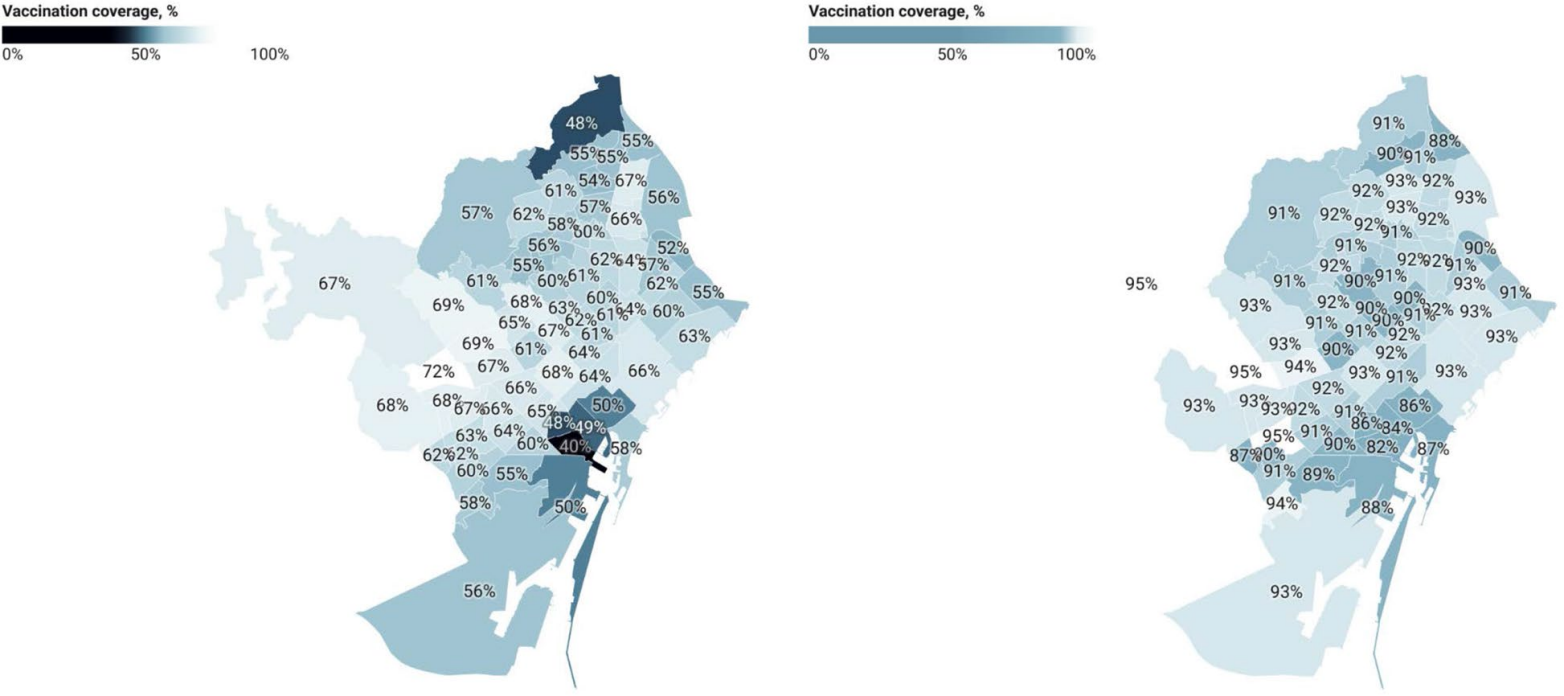
Vaccine coverage of the first dose of vaccine against COVID-19, in the group aged 60–69 years, on May 4, 2021 and November 30, 2021, by Basic Health Areas of the city of Barcelona

Vaccinations have been the key to change the course of the pandemic and lower the incidence of the disease. For people aged 70 years and older, primary healthcare (PHC) teams made an active personal appeal by telephone and vaccinated these persons in the same PHC centers: on June 15, this group had a coverage of over 90%, with a maximum range of 10 percentage points between the minimum and maximum of the 66 Basic Health Areas (BHA) of the city (a BHA is the geographic area assigned to a PHC team). However, to increase the system’s capacity and comply with distance and capacity restrictions, more vaccination points were opened, aimed at groups younger than 70 years. These groups are required to use an online self-appointment system. A few weeks after starting the first group, aged from 60 to 69 years, a gap in coverage of first dose was detected, from 40 to 72% across BHA (see Fig. 4), with lower coverage in the most deprived BHA. The same happened when starting younger age groups. Through a rapid consultation with community groups, we identified digital, language and cultural barriers as factors that could be influencing this delay in coverage. To this end, public health and healthcare services joined forces with municipal services of the districts with low coverage areas, to set up a network of 25 support points, aimed at helping people to schedule appointments, and later also 19 specific vaccination street points without scheduling the visit. In November, the range in coverage was 82 to 95% across BHA (Fig. 4).

## Discussion

### Socioeconomic inequalities in COVID-19 in Barcelona in the different waves

In a city that has been significantly affected by the COVID-19 pandemic, we have shown socioeconomic inequalities in the incidence of COVID-19, namely area-based income-related inequalities. This social gradient was more or less present in all four waves, being moderate in the first wave, based on hospitalized cases, largest in the second wave, especially for areas in the lowest income quintile, and almost absent in the two latter waves, with a significantly higher risk only for women in the lowest quintile neighborhoods. The daily online availability of these indicators has been an important surveillance tool to stimulate public policies to tackle inequalities.

Patterns of geographic and income-related inequalities in COVID-19 in the first and second waves in Barcelona have been described elsewhere [15–18], and a wider discussion on potential explanations has previously been reported [6]. The smaller inequalities in the first wave could be related to the insufficiency of diagnostic tests, reserved to healthcare workers and severe cases; thus the epidemic was underestimated more in some areas of the city with high levels of young immigrant population. When massive testing was undertaken, these areas appeared as those with the highest incidence, and inequalities linked to living and working conditions, such as job types (frontline jobs, fewer opportunities to work from home) [18, 19] and housing conditions (small dwellings, sometimes shared with other families) became evident [20–23]. The third and fourth waves started when several restrictions were lifted, e.g. on interprovince mobility and restaurant opening: it is likely that affluent classes experienced the most changes in social contacts from the lifting of restrictions, thus counterbalancing the factors described above and potentially resulting in smaller inequalities.

### Multisectoral equity-oriented policies to tackle the COVID-19 pandemic: lessons learned

The challenge of a pandemic that threatened to overwhelm the capacity of the health system mobilized the efforts of civil society and all sectors of local and regional government, which broke traditional boundaries to coordinate their forces in an exceptional moment. The sensitivity of many of these sectors to equity issues, and their willingness to respond to the needs of the most vulnerable groups detected through their contact with the health systems, together with the rapid availability of area-based data on the ASPB website, generated a political momentum in which policymakers asked public health officials to maximize efforts to develop policies to tackle these inequalities, coordinating actions in specific settings and enabling practical supports to equalize opportunities for self-isolation [24, 25]. Moreover, public health services made an effort to produce accessible and easy-to-read information about COVID-19 for professionals, decision-makers and citizens [26], although changing or delayed official information and uncertainties reflected in the media sometimes generated mistrust and/or misunderstandings [27].

All these policies required intense organizational and personal efforts, and rapid and creative action to address population needs as soon as they became evident. Naturally, the urgency to act resulted in many trial and error experiences, inefficiencies in resource use, and a limited possibility to systematically evaluate impacts.

## Conclusions

In this paper we have shown how COVID-19 hit Barcelona neighborhoods unequally, with variations between waves, and we have described a selection of targeted policies and collaborative interventions that helped to address the needs of the most disadvantaged populations. Policies did not eliminate socioeconomic inequalities in the incidence of COVID-19, but we suggest that they might have buffered the extent of inequalities and prevented them from being larger. Lastly, a community-based approach might have helped to tackle emerging inequalities in vaccination coverage by identifying and addressing digital and cultural barriers to online booking. Further studies would be needed to evaluate their actual impact on inequalities [28]. In Barcelona, several traditional limitations in information systems were overcome and surveillance methods will be reproducible in future epidemics or vaccination campaigns. The challenge now is to work to maintain those policies, working methods and priorities, as well as to tackle all social and economic inequalities due to the impacts of the pandemic.

## Data Availability

The datasets used and/or analyzed during the current study are available from the corresponding author on reasonable request. Some of the data are publicly available and visualized at the “#COVID19aldiaBCN” website: https://webs.aspb.cat/covid19aldiabcn/

## Abbreviations

RR: Relative risk
ASPB: *Agència de Salut Pública de Barcelona*
PHC: Primary healthcare
BHA: Basic Health Areas
